# Joint Task Offloading and Power Allocation for Satellite Edge Computing Networks

**DOI:** 10.3390/s25092892

**Published:** 2025-05-03

**Authors:** Yuxuan Li, Shibing Zhu, Ting Xiong, Yuwei Li, Qi Su, Jianmei Dai

**Affiliations:** School of Space Information, Space Engineering University, Beijing 101416, China; liyuxuan@hgd.edu.cn (Y.L.); zxbpaper@163.com (S.Z.); xiongting@bupt.edu.cn (T.X.); yuwei_li2022@163.com (Y.L.); suqi@hgd.edu.cn (Q.S.)

**Keywords:** satellite communication, multi-access edge computing, on-board edge computing, satellite edge computing network

## Abstract

Low Earth orbit (LEO) satellite networks have shown extensive application in the fields of navigation, communication services in remote areas, and disaster early warning. Inspired by multi-access edge computing (MEC) technology, satellite edge computing (SEC) technology emerges, which deploys mobile edge computing on satellites to achieve lower service latency by leveraging the advantage of satellites being closer to users. However, due to the limitations in the size and power of LEO satellites, processing computationally intensive tasks with a single satellite may overload it, reducing its lifespan and resulting in high service latency. In this paper, we consider a scenario of multi-satellite collaborative offloading. We mainly focus on computation offloading in the satellite edge computing network (SECN) by jointly considering the transmission power and task assignment ratios. A maximum delay minimization problem under the power and energy constraints is formulated, and a distributed balance increasing penalty dual decomposition (DB-IPDD) algorithm is proposed, utilizing the triple-layer computing structure that can leverage the computing resources of multiple LEO satellites. Simulation results demonstrate the advantage of the proposed solution over several baseline schemes.

## 1. Introduction

With the development of the Internet of Things, the proliferation of ubiquitous mobile devices is promoting many new applications and creating more new services [1]. In recent years, with the emergence of various new applications on user terminals, the requirement for low service latency is even more urgent. In order to process tasks more efficiently, users are requesting more computing resources from cloud centers. Although cloud computing can provide centralized access and powerful computing resources, due to the exponential growth of users’ computing demands the pressure on computing and data transmission for cloud computing is increasing. The unpredictable service latency experienced by users in remote regions, stemming from data transmission to distant cloud servers, often results in prohibitively high operational costs and failure to meet emerging applications’ real-time requirement [2]. As a remedy to these limitations, multi-access edge computing (MEC) technology came into being, and has attracted much research interest from both academia and industry [3,4,5]. It connects users with nearby computing facilities, providing low-latency offloading services for users. This not only reduces the service latency of users, but also greatly alleviates the computing pressure of cloud centers and the transmission pressure of backbone networks, thus becoming a new computing paradigm [6].

As the sixth-generation (6G) network continues to evolve, addressing the challenges of global coverage and ultra-low latency has become a pivotal focus for research and development. Terrestrial network edge computing can provide low-latency services for users at the edge of the network, provide feasible solutions for new applications and services spawned by IoT technology, and provide services with better data privacy protection [7,8]. However, the proportion of land covered by these services is less than 20% of the Earth’s surface, and the ground network only covers densely populated areas due to economic factors. For remote areas, such as oceans, deserts, and mountains, ground edge computing services are unavailable [9]. In addition, ground computing equipment is vulnerable to natural disasters such as earthquakes and fires, as well as human activities such as terrorist attacks and power failure. Therefore, to make up for the shortcomings of the terrestrial network, using aerial or satellite networks to provide services has become a promising solution.

Unlike aerial edge computing networks [10] that primarily rely on low-altitude platforms (e.g., unmanned aerial vehicles), satellite systems exhibit unique characteristics that demand tailored optimization frameworks. First, aerial networks prioritize optimizing UAV movement trajectories to dynamically minimize energy consumption under stringent onboard power constraints and rapid topology changes [11]. In contrast, satellite motion generally follows fixed orbital trajectories, allowing node positions to be calculated using orbital dynamics equations. Meanwhile, the high-speed movement of satellites induces non-negligible Doppler shifts, requiring Doppler effect mitigation in satellite edge computing frameworks—a challenge less emphasized in aerial computing systems. Furthermore, satellite networks face distinctive challenges such as intermittent inter-satellite link disruptions and periodic variations in solar power supply. These differences underscore the necessity of developing dedicated energy management strategies and latency-aware scheduling mechanisms for satellite edge computing, warranting in-depth exploration in system modeling and problem formulation.

Satellite edge computing (SEC) introduces MEC into satellite networks, which enables computational tasks to be performed in space. Satellite networks equipped with MEC are called a satellite edge computing network (SECN). The SECN not only provides low-latency network services for users in remote areas, but also serves as a supplement in densely populated areas when terrestrial networks cannot fulfill user requests. The SECN enables low-cost, seamless global coverage and has become one of the key technologies for the future 6G (sixth-generation) network [12,13].

The SECN overcomes the uneven distribution of terrestrial computing resources and the lack of network and computing services over the vast majority of the planet’s surface by shifting computing from the ground to space [14]. Because they can achieve seamless global coverage, users can access a variety of services provided by the SECN, including edge computing resources and wireless storage resources, no matter where they are in the world. Users in remote regions do not need to apply for computing and network services from remote cloud centers, and the SECN can greatly reduce queuing and transmission latencies, thereby improving the quality of service [15]. For the cloud center and backbone network, the SECN can share the processing load of numerous tasks, and data do not have to be transmitted through the backbone network, reducing the workload of both. Finally, the SECN processes the data in the satellite MEC in a distributed approach, which greatly reduces the risk of data leakage and improves the security of user data [16].

**Figure 1:**
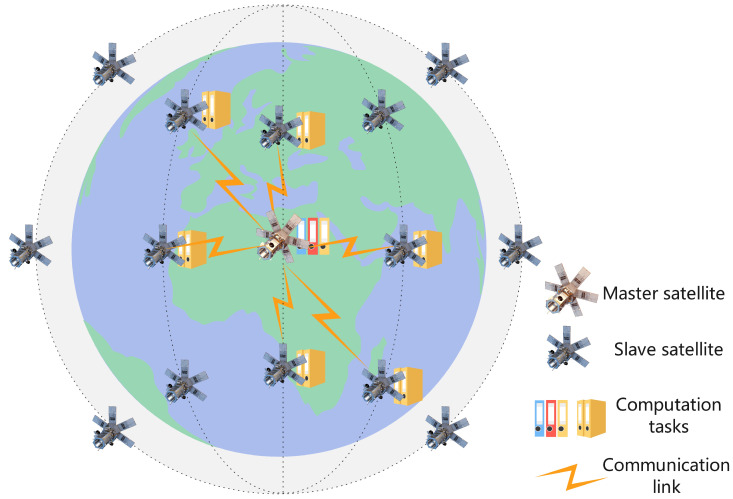
Illustration of the LEO satellite edge computing system. Each satellite has the capability to participate in task offloading and computation, while being subject to resource constraints. During data processing, the data transmission between the MS and the SS occurs over wireless links.

Although the SECN can improve the user experience and reduce the workload of the network, existing studies only consider the scenario in which each satellite handles a task independently, without considering the complex case when some tasks are too heavy to be handled by a single satellite [17,18]. In such a situation, excessive satellite load happens, which reduces the service lifespan of the satellite and causes a waste of valuable resources. Cooperative satellite computing provide a new solution to this challenge. As shown in Figure 1, this paper considers a multi-satellite cooperative satellite edge computing model where multiple satellites compute in parallel to reduce the maximum latency. The maximum delay minimization problem in the proposed scenario is nonconvex and high-coupled. We consider using the increasing penalty dual decomposition (IPDD) algorithm to minimize the maximum delay of the system under the power and energy constraints, where the IPDD algorithm is a double-loop iterative algorithm that can solve nonconvex nonsmooth optimization problems, especially when the optimization variables are nonlinearly coupled in some nonconvex constraints [19]. While building upon the established IPDD algorithm framework, we present systematic enhancements specifically optimized for latency minimization in satellite edge computing architectures named delay-balanced increasing penalty dual decomposition (DB-IPDD). Our methodological innovations specifically address the unique constraints of multi-satellite collaborative environments. We developed a novel analytical framework that establishes Theorem 1 through rigorous proof-by-contradiction methodology, formally demonstrating that system-wide latency minimization occurs under uniform processing time distribution across satellite nodes. Through evaluating the optimal solution obtained via the IPDD method, we can verify whether it satisfies the optimality criteria established in Theorem 1. If the criteria are not met, we iteratively optimize the resource allocation to balance the processing times between the satellites with the maximum and minimum latencies. This process is repeated until convergence is achieved.

This DB-IPDD method ensures progressive refinement toward global optimality, representing a substantial advancement over conventional IPDD implementations. The theoretical foundation established through Theorem 1’s proof, combined with our adaptive resource balancing protocol, provides a replicable framework for latency-sensitive distributed space systems.

The main contributions are as follows:

- We construct a multi-satellite SECN scenario, in which a single task is assumed to be divided into sub-tasks and offloaded to adjacent satellites. These sub-tasks will be processed on board. This offloading strategy makes full use of heterogeneous resources in the satellite network, reduces the load burden on individual satellites, and enables a more flexible resource scheduling scheme.
- We formulate an optimization problem mathematically, to minimize the maximum delay through resource allocation, in which transmission power and task assignment ratios are optimized.
- We propose a distributed balance increasing penalty dual decomposition (DB-IPDD) algorithm. Our experimental validation across various orbital configurations demonstrates consistent 14.3% latency reduction compared with baseline approaches.
- We derive the optimal solution theoretically for the LEO satellite case, in which the resources are abundantly available. We further extend the analysis to the general case under specific conditions.
- Simulation results clearly show that the proposed solution greatly outperforms the baseline methods, and the transmission delay is significantly reduced.

The rest of the paper is organized as follows. Section 2 introduces the current research and development status related to LEO satellite edge computing. Section 3 provides a depiction of the distributed satellite edge computing network system model and the resource allocation problem. In Section 4, we introduce the algorithm for slave satellite selection, data offloading, and transmission power allocation. Simulation results of the proposed algorithm are presented in Section 5. Finally, the paper is concluded in Section 6.

## 2. Related Work

With the development of satellite communication, the on-board computing power of satellites has increased rapidly and the launch cost of satellites has decreased gradually, contributing to the feasibility of the SECN. Inspired by the advantages of the SECN over traditional satellite networks, many efforts have been put into both the architecture and real-world industrial applications of the SECN in the past few years. In this study, we briefly review the relevant literature and divide the literature into two categories, namely, SECN task offloading strategy design, which aims to find the appropriate offloading strategy under different optimization objective functions and constraints, and SECN architecture design, which optimizes the SECN architecture and working mode to improve network performance.

### 2.1. Task Offloading Strategy Design

Considering the fine-grained energy utilization in order to prolong the useful life of satellites, an iterative algorithm is proposed in [20] to minimize the system weighted energy consumption while satisfying latency requirements in task offloading. This scheme saves a significant portion of system energy consumption compared with other offloading schemes. A greedy task allocation algorithm is proposed in [21] for SEC performing in the Walker satellite constellation, where each task is uniquely selected to reduce the average cost of task computing. A mechanism is designed in [22] that maximizes the network performance by jointly optimizing the computation offloading scheme, transmission scheduling discipline, and a pricing rule. A distributed computing offloading method based on game theory is proposed in [23]. This method enables mobile device users to improve the overall performance of the network through the trade-off between local computing and offloading to the edge server. It also involves non-cooperative game interaction with other users when offloading computations occur between multi-user mobile devices and edge servers. A multi-hop satellite peer offloading (MHSPO) algorithm is proposed in [24] for the time-varying satellite network topology. This algorithm solves the problem of how to achieve efficient offloading cooperation under the conditions of limited resources, thus further improving service quality and resource utilization.

### 2.2. SECN Architecture Design

Taking into account the design of a multi-tier satellite edge computing framework and the synchronized scheduling of diverse edge computing resources, virtual network functions (VNFs) are deployed in the SECN to achieve low-latency network services by jointly optimizing the placement of VNFs and the routing of traffic under the capacity constraints of LEO satellites and the budget constraints of each NFV-enabled request [1]. Not only is the case of individual user requests considered, but also the uncertainty and dynamics of user requests in the online environment are taken into account. A SECN architecture with three layers, which includes GEO, LEO, and ground layers, is designed in [25]. The high-speed movement of LEO satellites is considered and an ADMM-based distributed optimization algorithm is proposed to solve the mobility-aware computation offloading problem in the SECN. An IoT supportable SECN architecture is proposed in [26], and a heuristic algorithm with low time complexity is proposed to reduce the weighted sum of latency, computational power, and transmission power attenuation. To address limited computing resources in remote satellite–terrestrial networks, a double-edge computing offloading algorithm that effectively reduces task processing delay and energy consumption by optimizing offloading to satellite and terrestrial edge nodes is proposed in [27].

## 3. System Model and Problem Formulation

### 3.1. System Model

The LEO satellite edge computing system is shown in Figure 1. We denote the satellite set as M={0,1,2,…,M}, which contains M+1 LEO satellites equipped with MEC servers. In this scenario, there is one master satellite (MS) that generates the computation task, and the remaining LEO satellites in the communication range are slave satellites (SSs), which receive and assist in processing the computation task data. When the MS generates a time-sensitive task and there are not enough computing resources in the MS, part of the data generated by the task can be offloaded to the SSs in a cooperative approach. We assume that the time period *T* is divided into *N* slots, which are denoted by i∈T={1,2,…,N}. In this time slot, *i*, the physical network topology of the satellite system is assumed to be fixed and the set of SSs is represented by k∈K={1,2,…,M}. All parameters used in this study are summarized and shown in Table 1.

### 3.2. Doppler Frequency Shift Model

We assume that all orbital elements (Semi-major Axis, Eccentricity, Inclination, Longitude of the ascending node, Argument of periapsis, and Mean Anomaly) of the LEO satellite are known to the users, which are represented as [a,e,i,ω,Ω,φ]. The orbital coordinate system can be transformed into a central celestial inertial system after cubic direction cosine matrix transformation, and the position vector of the LEO satellite can be formulated as

(1) $$\begin{array}{cc} r & =R_{3}\left(-\Omega \right)R_{1}\left(-i\right)R_{3}\left(-\omega \right)\left[\begin{array}{c} r\cos\varphi \\ r\sin\varphi \\ 0 \end{array}\right]\\ & =\frac{p}{1+e\cos\varphi }\left[\begin{array}{c} \cos\Omega \cos(\omega +\varphi )-\sin\Omega \sin(\omega +\varphi )\cos i\\ \sin\Omega \cos(\omega +\varphi )+\cos\Omega \sin(\omega +\varphi )\cos i\\ \sin(\omega +\varphi )\sin i \end{array}\right], \end{array}$$

where *R* denotes the rotation matrix, and *p* and *r* represent the semi-latus rectum of the orbit, which satisfies $p=a(1-e^{2})$ and the size of the position vector $r=\frac{p}{1+e\cos\varphi }$, respectively.

The velocity vector of the LEO satellite can be obtained by differentiating the position vector against variable φ. The expression of the velocity vector is shown as

(2) $$\begin{array}{cc} v & =\sqrt{\frac{\mu }{p}}\left[\begin{array}{c} -\cos\Omega (\sin(\omega +\varphi )+e\sin\omega )-\sin\Omega (\cos(\omega +\varphi )+e\cos\omega )\cos i\\ -\sin\Omega (\sin(\omega +\varphi )+e\sin\omega )+\cos\Omega (\cos(\omega +\varphi )+e\cos\omega )\cos i\\ (\cos(\omega +\varphi )+e\cos\omega )\sin i \end{array}\right]. \end{array}$$

The motion of the LEO satellite generates the Doppler frequency shift. When the MS offloads to SSs, they initiate requests to each other and exchange their location information and velocity vectors. Subsequently, the SSs calculate the Doppler shift based on their relative velocity and position, and apply the necessary compensation to mitigate the challenges of demodulation at the satellite.

The frequency received in the SSs can be calculated by

(3) $$\begin{array}{c} f_{SS}=\frac{c+v_{\mathrm{SS}}\cdot r}{c+v_{\mathrm{MS}}\cdot r}f_{MS}, \end{array}$$

The Doppler frequency shift of the SSs can be denoted by

(4) $$\begin{array}{cc} f_{d} & =f_{SS}-f_{MS} \end{array}$$

(5) $$\begin{array}{cc} \; & =\frac{v_{\mathrm{SS}}\cdot r-v_{\mathrm{MS}}\cdot r}{c+v_{\mathrm{MS}}\cdot r}f_{MS}, \end{array}$$

where r denote the unit vector in the direction of signal propagation (from the MS to the SSs), $v_{\mathrm{SS}}$ and $v_{\mathrm{MS}}$ denote the velocity vector of the SSs and MS, respectively, and $f_{MS}$ represents the carrier frequency of the MS.

### 3.3. Task Offloading Model

The wireless channel between satellites is predominantly Line-of-Sight (LOS). The channel gain from the *k*-th SS to the MS in the *i*-th time slot is denoted by $h_{k,i}$, given as follows:

(6) $$\begin{array}{c} h_{k,i}=\mu_{0}d_{k,i}^{-2}\;k\in K,i\in T, \end{array}$$

where $\mu_{0}$ is the channel power gain at a reference distance $d_{0}=1m$, and the distance between the *k*-th SS with the MS at the *i*-th time slot is denoted by $d_{k,i}$.

We define $\alpha_{k,i}$ as the ratio of data offloaded to satellite *k* in time slot *i*,

(7) $$\begin{array}{c} 0\leq \alpha_{k,i}\leq 1,\;k\in K,i\in T. \end{array}$$

The transmission power of the MS to the *k*-th SS in the *i*-th time slot is denoted as $P_{k,i}$. $R_{k,i}$ is denoted as the normalized transmission data rate from the MS to the *k*-th SS in the time slot *i*:

(8) $$\begin{array}{c} R_{k,i}=\log_{2}\left(1+\frac{P_{k,i}h_{k,i}}{\sigma^{2}}\right), \end{array}$$

where $\sigma^{2}$ denotes the Additive White Gaussian Noise (AWGN) power, which we assume to be a constant.

We assume the task in the *i*-th time slot generates $D_{i}$ bits of data. The computation task remaining in the local can be expressed as $\alpha_{0,i}D_{i}$.

$C_{k}$ denotes the main frequency of the MEC server in the *k*-th SS, which is the number of CPU cycles per second, $C_{0}$ denotes the main frequency of the MEC server in the MS, and *s* denotes the number of cycles of CPU to execute one bit of data. Thus, the local computation time, $t_{0,i}$, is denoted as

(9) $$\begin{array}{c} t_{0,i}=\frac{\alpha_{0,i}D_{i}s}{C_{0}}. \end{array}$$

The computing time of slave satellite *k* in the *i*-th time slot can be expressed as $\alpha_{k,i}D_{i}s/C_{k}$. The transmission delay of slave satellite *k* in the *i*-th time slot can be expressed as $\alpha_{k,i}D_{i}/R_{k,i}B+2d_{k,i}/c$, where *B* represents the bandwidth of the channel and *c* represents the speed of free-space light.

Combining the above expressions, we can obtain the total task completion time, $t_{k,i}$, as:

(10) $$\begin{array}{c} t_{k,i}=\frac{\alpha_{k,i}D_{i}s}{C_{0}}+\frac{\alpha_{k,i}D_{i}}{R_{k,i}B}+2\frac{d_{k,i}}{c}. \end{array}$$

#### Energy Consumption Model

The energy consumption of the MS can be expressed as [28]:

(11) $$\begin{array}{c} E_{i}^{master}=r_{m}\alpha_{0,i}D_{k,i}C_{0}^{2}, \end{array}$$

where $r_{m}$ is the effective capacitance coefficient of the chip equipped in the MEC server of the MS.

The transmission consumption in the offloading procedure from the MS to the *k*-th slave satellite can be presented as

(12) $$\begin{array}{c} E_{i}^{trans}=\frac{P_{k,i}\alpha_{k,i}D_{k,i}}{R_{k,i}B} \end{array}$$

The energy consumption of the *k*-th SS can be expressed as

(13) $$\begin{array}{c} E_{k,i}^{slave}=r_{k}\alpha_{k,i}D_{k,i}C_{0}^{2}, \end{array}$$

in the same way, $r_{s}$ is the effective capacitance coefficient of the chip of the *k*-th slave satellite.

### 3.4. Resource Constraints

We denote $E_{1}$ as the upper limit of energy consumption for completing a data processing task among all participating LEO satellites, while $E_{2}$ represents the upper limit of energy consumption for any individual satellite involved in the task processing. $P_{m}$ denotes the maximum operating power of the satellite. The energy and power constraints can be expressed as follows:

(14) $$\begin{array}{ccc} \; & \sum_{i=1}^{N}r_{m}C_{0}^{2}D_{i}\alpha_{0,i}+\sum_{i=1}^{N}\sum_{k=1}^{M}\frac{P_{k,i}\alpha_{k,i}D_{i}}{R_{k,i}B}\leq E_{1} & \end{array}$$

(15) $$\begin{array}{ccc} \; & \sum_{i=1}^{N}r_{k}\alpha_{k,i}D_{i}C_{k}^{2}\leq E_{2} & \end{array}$$

(16) $$\begin{array}{ccc} \; & \sum_{i=1}^{M}P_{k,i}+r_{m}C_{0}^{3}\leq P_{m} & , \end{array}$$

where constraint (14) ensures that the total energy consumption is smaller than E, constraint (16) indicates that the maximum power of the MS is less than $P_{m}$, and constraint (33) expresses that the maximum working power of the SS is not higher than $P_{s}$.

### 3.5. Problem Formulation

The offloading delay includes transmission delay and computation delay, which are presented in (9) and (10) separately. To minimize the sum of the offloading delay in the period *T* which contains *N* slots, the optimization problem can be expressed as follows:

(17) $$\begin{array}{cc} \; & \min_{\alpha_{k,i},P_{k,i},\kappa_{k,i}}\sum_{i=1}^{N}\max_{\forall k\in M}t_{k,i} \end{array}$$

(18) $$\begin{array}{c} s.t.0\leq \sum_{k=1}^{M}\alpha_{k,i}\leq 1 \end{array}$$

(19) $$\begin{array}{cc} \; & 0\leq \alpha_{k,i}\leq 1,\;k\in K,i\in T. \end{array}$$

(20) $$\begin{array}{cc} & R_{k,i}=\log_{2}\left(1+\frac{P_{k,i}h_{k,i}}{\sigma^{2}}\right), \end{array}$$

(21) $$\begin{array}{cc} & t_{0,i}=\frac{\alpha_{0,i}D_{i}s}{C_{0}}, \end{array}$$

(22) $$\begin{array}{cc} & t_{k,i}=\frac{\alpha_{k,i}D_{i}s}{C_{0}}+\frac{\alpha_{k,i}D_{i}}{R_{k,i}B}+2\frac{d_{k,i}}{c} \end{array}$$

(23) $$\begin{array}{ccc} & \sum_{i=1}^{N}r_{m}C_{0}^{2}D_{i}\alpha_{0,i}+\sum_{i=1}^{N}\sum_{k=1}^{M}\frac{P_{k,i}\alpha_{k,i}D_{i}}{R_{k,i}B}\leq E_{1} & \end{array}$$

(24) $$\begin{array}{ccc} \; & \sum_{i=1}^{N}r_{k}\alpha_{k,i}D_{i}C_{k}^{2}\leq E_{2} & \end{array}$$

(25) $$\begin{array}{ccc} \; & \sum_{i=1}^{M}P_{k,i}+r_{m}C_{0}^{3}\leq P_{m} & . \end{array}$$

In order to eliminate denominator variables, we introduce auxiliary variables and derive a new expression. Here, $\kappa_{k,i}$ represents the reciprocal of $R_{k,i}$ based on the initial problem formulation:

(26) $$\begin{array}{c} \kappa_{k,i}\log_{2}\left(1+\frac{P_{k,i}\mu_{0}}{\sigma^{2}d^{2}}\right)=1 \end{array}$$

The reformulated problem is as follows:

(27) $$\begin{array}{cc} \; & \min_{\alpha_{k,i},P_{k,i},\kappa_{k,i}}\sum_{i=1}^{N}\max_{\forall k\in M}t_{k,i} \end{array}$$

(28) $$\begin{array}{ccc} s.t.\; & t_{k,i}=\frac{\alpha_{k,i}D_{i}s}{C_{0}}+\frac{\alpha_{k,i}\kappa_{k,i}D_{i}}{B}+2\frac{d_{k,i}}{c} & \end{array}$$

(29) $$\begin{array}{cc} \; & t_{0,i}=\frac{\alpha_{0,i}D_{i}s}{C_{0}} \end{array}$$

(30) $$\begin{array}{cc} \; & \sum_{i=1}^{N}r_{m}C_{0}^{2}D_{i}\alpha_{0,i}+\sum_{i=1}^{N}\sum_{k=1}^{M}\frac{P_{k,i}\alpha_{k,i}D_{i}\kappa_{k,i}}{B}\leq E_{1} \end{array}$$

(31) $$\begin{array}{cc} & 0\leq \alpha_{k,i}\leq 1,\;k\in K,i\in T. \end{array}$$

(32) $$\begin{array}{cc} & R_{k,i}=\log_{2}\left(1+\frac{P_{k,i}h_{k,i}}{\sigma^{2}}\right), \end{array}$$

(33) $$\begin{array}{cc} \sum_{i=1}^{N}r_{k}\alpha_{k,i}D_{i}C_{k}^{2}\leq E_{2} & \end{array}$$

(34) $$\begin{array}{cc} \; & \sum_{i=1}^{M}P_{k,i}+r_{m}C_{0}^{3}\leq P_{m}, \end{array}$$

(35) $$\begin{array}{cc} & \kappa_{k,i}\log_{2}\left(1+\frac{P_{k,i}\mu_{0}}{\sigma^{2}d^{2}}\right)=1. \end{array}$$

Nevertheless, there are still a significant number of equality constraints and coupling variables. Traditional methods like gradient descent often struggle to achieve satisfactory results when dealing with such nonconvex optimization problems.

The increasing penalty dual decomposition (IPDD) algorithm excels in handling the complexity of nonconvex problems and efficiently optimizing the solutions by combining the penalty method and the Alternating Lagrangian (AL) method [16]. However, the IPDD algorithm cannot take advantage of the distributed structural characteristics, and cannot obtain the globally optimal solution.

## 4. Resource Management Algorithm

Inspired by the IPDD algorithm, we propose a new resource management algorithm called DB-IPDD, which features a triple-loop structure. In the inner loop, we optimize primary variables by solving the AL sub-problem. In the middle loop, we reallocate the resources based on Theorem 1. In the outer loop, the dual variables and penalty parameters are updated. The algorithm terminates when the errors of the equation constraints become sufficiently small or the maximum number of iterations is reached.

We introduce auxiliary variables $W_{k,i}$ and $V_{k,i}$ to replace coupling variables $\alpha_{k,i}\kappa_{k,i}$ and $P_{k,i}\kappa_{k,i}\alpha_{k,i}$, respectively, and incorporate the equality constraints into the objective function as penalty terms. After that, a new objective function is constructed as follows.

(36) $$\begin{array}{cc} P_{1}: & \min_{X}\sum_{i=1}^{N}\max_{1\leq k\leq M}t_{k,i}+P_{\rho }\left(X\right) \end{array}$$

(37) $$\begin{array}{cc} s.t. & 0\leq \alpha_{k,i}\leq 1,\;k\in K,i\in T. \end{array}$$

(38) $$\begin{array}{cc} \; & \sum_{i=1}^{N}r_{k}\alpha_{k,i}D_{i}C_{k}^{2}\leq E_{2} \end{array}$$

(39) $$\begin{array}{cc} \; & \sum_{i=1}^{N}r_{m}C_{0}^{2}D_{i}\alpha_{0,i}+\sum_{i=1}^{N}\sum_{k=1}^{M}\frac{P_{k,i}\alpha_{k,i}D_{i}\kappa_{k,i}}{B}\leq E_{1} \end{array}$$

(40) $$\begin{array}{cc} \; & \sum_{i=1}^{M}P_{k,i}+r_{m}C_{0}^{3}\leq P_{m} \end{array}$$

where $P_{\rho }\left(X\right)$ can be shown as

(41) $$\begin{array}{cc} P_{\rho }\left(X\right)= & \frac{1}{2\rho }\sum_{i=1}^{N}\sum_{k=1}^{M}{\left(t_{k,i}-\frac{D_{i}s}{C_{0}}\alpha_{k,i}-\frac{D_{i}}{B}W_{k,i}+\rho \lambda_{k,i}^{1}\right)}^{2}+\\ & \frac{1}{2\rho }\sum_{i=1}^{N}{\left(t_{0,i}-\frac{D_{i}s}{C_{0}}\alpha_{0,i}+\rho \lambda_{0,i}^{1}\right)}^{2}+\\ \; & \frac{1}{2\rho }\sum_{i=1}^{N}\sum_{k=1}^{M}{\left(V_{k,i}-P_{k,i}\kappa_{k,i}\alpha_{k,i}+\rho \lambda_{k,i}^{2}\right)}^{2}+\\ \; & \frac{1}{2\rho }\sum_{i=1}^{N}\sum_{k=1}^{M}{\left(W_{k,i}-\alpha_{k,i}\kappa_{k,i}+\rho \lambda_{k,i}^{3}\right)}^{2}+\\ \; & \frac{1}{2\rho }\sum_{i=1}^{N}\sum_{k=1}^{M}{\left(\kappa_{k,i}\log_{2}\left(1+\frac{P_{k,i}\mu_{0}}{\sigma^{2}d^{2}}\right)-1+\rho \lambda_{k,i}^{4}\right)}^{2}. \end{array}$$

The overall procedure is outlined as follows.

### 4.1. Inner Loop Procedure

We optimize primary variables separately by exploiting the block structure in the inner loop, thereby avoiding the challenging process of optimizing all variables simultaneously. In this paper, we optimize a single variable while other variables are fixed by using a block coordinate descent-type method. In each block, we solve the first-order optimality condition by setting the gradient of the objective equal to zero. Then, we project the obtained solution onto the variable set. If the solution falls within the range of the variable set, it becomes the variable update value. Otherwise, the variable update value should be a point in the variable set that is closest to the original solution.

The following is the solving procedure for α, *P*, κ, *W*, *V*, λ, and ρ.

1. Initial values of α, *P*, κ, *W*, *V*, λ, and ρ are set to $\alpha^{0}$, $P^{0}$, $\kappa^{0}$, $W^{0}$, $V^{0}$, $\lambda^{0}$, and $\rho^{0}$, respectively; then, set k=1 and i=1.
2. Optimize $W_{k,i}$ by *W*-sub-problem (42a), which is a quadratic unconstrained optimization problem.
  (42a) $$\begin{array}{cc} P_{W}:\min_{W_{k,i}} & \sum_{i=1}^{N}\sum_{k=1}^{M}{\left(t_{k,i}-\frac{D_{i}s}{C_{0}}\alpha_{k,i}-\frac{D_{i}}{B}W_{k,i}+\rho \lambda_{k,i}^{1}\right)}^{2}+\\ & \sum_{i=1}^{N}\sum_{k=1}^{M}{\left(W_{k,i}-\alpha_{k,i}\kappa_{k,i}+\rho \lambda_{k,i}^{3}\right)}^{2} \end{array}$$
  (42b) $$\begin{array}{c} s.t.W_{k,i}\geq 0 \end{array}$$
3. Optimize $\alpha_{k,i}$ by (43a), which is also a quadratic optimization problem.
  (43a) $$\begin{array}{cc} P_{\alpha }:\min_{\alpha_{k,i}} & \sum_{i=1}^{N}\sum_{k=1}^{M}{\left(t_{k,i}-\frac{D_{i}s}{C_{0}}\alpha_{k,i}-\frac{D_{i}}{B}W_{k,i}+\rho \lambda_{k,i}^{1}\right)}^{2}+\\ & \sum_{i=1}^{N}{\left(t_{0,i}-\frac{D_{i}s}{C_{0}}\alpha_{0,i}+\rho \lambda_{0,i}^{1}\right)}^{2}+\\ & \sum_{i=1}^{N}\sum_{k=1}^{M}{\left(V_{k,i}-P_{k,i}\kappa_{k,i}\alpha_{k,i}+\rho \lambda_{k,i}^{2}\right)}^{2}+\\ & \sum_{i=1}^{N}\sum_{k=1}^{M}{\left(W_{k,i}-\alpha_{k,i}\kappa_{k,i}+\rho \lambda_{k,i}^{3}\right)}^{2} \end{array}$$
  (43b) $$\begin{array}{cc} s.t. & \sum_{i=1}^{N}r_{m}C_{0}^{2}D_{i}\alpha_{0,i}+\sum_{i=1}^{N}\frac{P\cdot \alpha_{k,i}D}{R\cdot B}\leq E_{1} \end{array}$$
  (43c) $$\begin{array}{cc} \; & \sum_{i=1}^{N}\sum_{k=1}^{M}r\alpha_{k,i}D_{i}C_{0}^{2}\leq E_{2} \end{array}$$
4. Optimize $P_{k,i}$ by (44a).
  (44a) $$\begin{array}{c} P_{P}:\min_{P_{k,i}}\sum_{i=1}^{N}\sum_{k=1}^{M}{\left(V_{k,i}-P_{k,i}\kappa_{k,i}\alpha_{k,i}+\rho \lambda_{k,i}^{2}\right)}^{2}+\\ \sum_{i=1}^{N}\sum_{k=1}^{M}{\left(\kappa_{k,i}\log_{2}\left(1+\frac{P_{k,i}\mu_{0}}{\sigma^{2}d^{2}}\right)-1+\rho \lambda_{k,i}^{4}\right)}^{2} \end{array}$$
  (44b) $$\begin{array}{c} s.t.P_{k,i}\leq Pm-\sum_{m\neq k}P_{k,i}-r_{m}C_{0}^{3} \end{array}$$
5. Optimize $V_{k,i}$ by (45a).
  (45a) $$\begin{array}{c} P_{V}:\min_{V_{k,i}}\sum_{i=1}^{N}\sum_{k=1}^{M}{\left(V_{k,i}-P_{k,i}\kappa_{k,i}\alpha_{k,i}+\rho \lambda_{k,i}^{2}\right)}^{2} \end{array}$$
  (45b) $$\begin{array}{c} s.t.V_{k,i}\geq 0 \end{array}$$
6. Optimize $\kappa_{k,i}$ by (46a).
  (46a) $$\begin{array}{c} P_{\kappa }:\min_{\kappa_{k,i}}\sum_{i=1}^{N}\sum_{k=1}^{M}{\left(V_{k,i}-P_{k,i}\kappa_{k,i}\alpha_{k,i}+\rho \lambda_{k,i}^{2}\right)}^{2}\\ +\sum_{i=1}^{N}\sum_{k=1}^{M}{\left(W_{k,i}-\alpha_{k,i}\kappa_{k,i}+\rho \lambda_{k,i}^{3}\right)}^{2}\\ +\sum_{i=1}^{N}\sum_{k=1}^{M}{\left(\kappa_{k,i}\log_{2}\left(1+\frac{P_{k,i}\mu_{0}}{\sigma^{2}d^{2}}\right)-1+\rho \lambda_{k,i}^{4}\right)}^{2} \end{array}$$
  (46b) $$\begin{array}{c} s.t.\sum_{i=1}^{N}r_{m}C_{0}^{2}D_{i}\alpha_{0,i}+\frac{1}{B}\sum_{i=1}^{N}\sum_{k=1}^{M}P_{k,i}\alpha_{k,i}\kappa_{k,i}D_{i}\leq E_{1} \end{array}$$

### 4.2. Middle Loop Procedure

**Theorem** **1.**  *When $t_{0,i}=t_{1,i}=\cdots =t_{M,i}$, the minimum delay of the system in the $i_{\mathrm{th}}$ slot is achieved.*

**Proof.**  Details are given in Appendix A.1.    □

According to Theorem 1, we further reduce delay by reducing the data offloading ratio for the SS with the highest delay while increasing its transmission power, and by increasing the data offloading ratio for the SS with the lowest delay while decreasing its transmission power.

We denote $\alpha_{max}$ and $P_{max}$ as the data offloading ratio and transmission power of the SS with the highest delay, and $\alpha_{min}$ and $P_{min}$ as the data offloading ratio and transmission power of the SS with the lowest delay. Then we have   

(47a) $$\begin{array}{c} \alpha_{max}=\alpha_{max}-\Delta_{1} \end{array}$$

(47b) $$\begin{array}{c} P_{max}=P_{max}+\Delta_{2} \end{array}$$

(47c) $$\begin{array}{c} \alpha_{min}=\alpha_{min}+\Delta_{1} \end{array}$$

(47d) $$\begin{array}{c} P_{min}=P_{min}-\Delta_{2} \end{array}$$

where $\Delta_{1}$ and $\Delta_{2}$ are constants greater than zero.

### 4.3. Outer Loop Procedure

Once the AL sub-problems are approximately solved, the dual variables and the penalty parameters are updated in the outer loop. We introduce the constraint violation indicator function $h(Z^{n})$, which measures the violation of equality constraints in the *n*th iteration.

(48) $$\begin{array}{cc} h\left(Z_{i}\right)= & [t_{k,i}-\frac{D_{i}\cdot \alpha_{k,i}}{sC_{0}}-\frac{D_{i}\cdot W_{k,i}}{B},\\ \; & t_{0,i}-\frac{D_{i}\cdot \alpha_{0,i}s}{C_{0}},\\ \; & P_{k,i}\cdot \kappa_{k,i}\cdot \alpha_{k,i}-V_{k,i},\\ \; & \alpha_{k,i}\cdot \kappa_{k,i}-W_{k,i},\\ \; & \kappa_{k,i}\cdot \log_{2}\left(1+\frac{P_{k,i}\mu_{0}}{\sigma^{2}d^{2}}\right)-1] \end{array}$$

**Figure 2:**
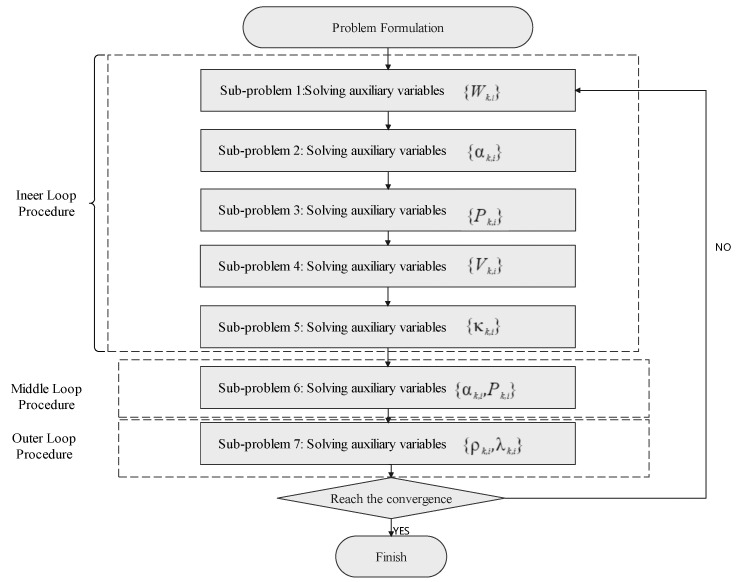
The flowchart of the DB-IPDD algorithm.

If the indicator satisfies $∥h\left(Z^{n}\right)∥\geq \eta_{k}$ or the loop number is less than the maximum loop number *N*, the AL multipliers and penalty factors $\lambda_{k,i}$, $\rho_{k,i}$ are updated by the following formulas:

(49a) $$\begin{array}{ccc} \; & \lambda_{k,i}^{n}=\lambda_{k,i}^{n-1}+\frac{1}{\rho_{k}}\left(h\left(Z_{i}\right)\right) & \forall k,i \end{array}$$

(49b) $$\begin{array}{ccc} \; & \rho_{i}^{n}=\gamma \rho_{i}^{n-1} & \forall i \end{array}$$

where γ is a constant between 0 and 1. It is easy to see that, when $h(Z^{n})=0$, the problem is solved to a Karush–Kuhn–Tucker (KKT) stationary solution. After *n* iterations of the algorithm, the violation indicator will approach some threshold, ζ, and if ζ≤η (where η is defined accuracy threshold), we obtain the approximate KKT solution of the problem. The flowchart and pseudocode of the proposed DB-IPDD algorithm are shown in Figure 2 and Algorithm 1, respectively.   
**Algorithm 1:** DB-IPDD Algorithm
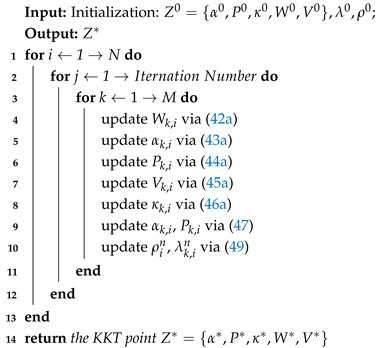


### 4.4. Time Complexity of DB-IPDD

Since the number of the optimized variables is NMK, the complexity of the inner loop procedure is O(NMK). For the middle procedure, the complexity of the procedure is O(2NM). For the outer loop procedure, the complexity can be denoted by O(2NM). As a result, the total complexity of the DB-IPDD algorithm can be given as O(INM(K+4)), where *I* is the number of iterations. The DB-IPDD algorithm achieves low time complexity as polynomial for the data size and also low time complexity with respect to the number of satellites for large-scale satellite networks.

## 5. Performance Analysis

**Figure 3:**
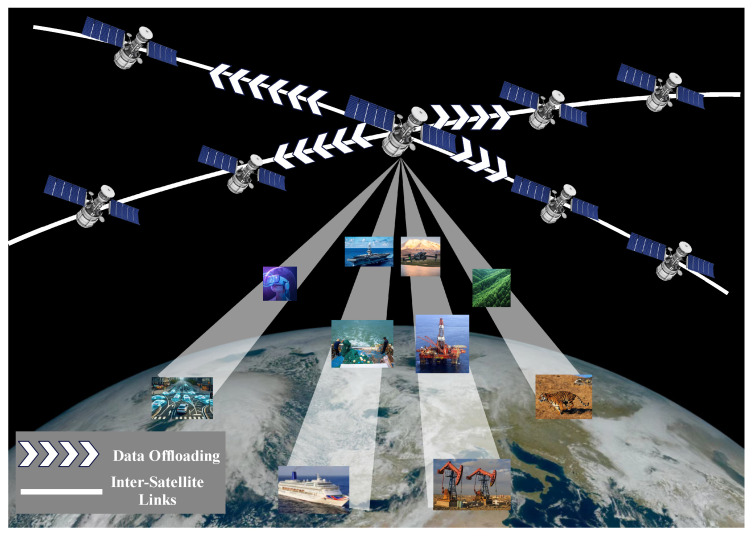
The distribution map of satellites in the scenario.

To verify the performance of the algorithm, we consider a scenario with one MS and four SSs serving a time-sensitive task. The distance between each SS and the MS is generated randomly. The AWGN power is set to $\sigma^{2}=1\times 10^{-8}$ W. We assume that all satellites have the same computing capability. The default values of typical parameters are listed in Table 2 and the distribution map of satellites in the scenario is shown in Figure 3.

### 5.1. Delay Performance

Regarding the performance verification of the DB-IPDD algorithm, we compare the proposed task offloading and resource allocation algorithm with the following algorithms: (1) Alternative Optimization (AO) algorithm; (2) Genetic algorithm; (3) Greedy algorithm; and (4) IPDD algorithm, where all schemes are running in the same simulation environment.

**Figure 4:**
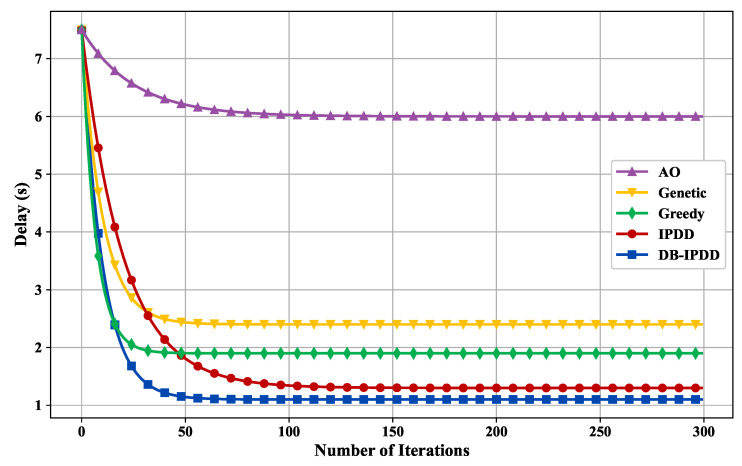
The delay of different schemes with a varying number of iterations.

The convergence of the DB-IPDD algorithm and the delay performance under different resource allocation schemes are verified in Figure 4. We can observe that the DB-IPDD algorithm has good convergence. The performance of the DB-IPDD and IPDD algorithms is obviously better than that of the Greedy, Genetic, and AO algorithms. The performance of the DB-IPDD algorithm is better than that of IPDD, which is because the DB-IPDD adopts Theorem 1 to find a better resource allocation condition.

### 5.2. Transmit Power Impact on Delay Performance

**Figure 5:**
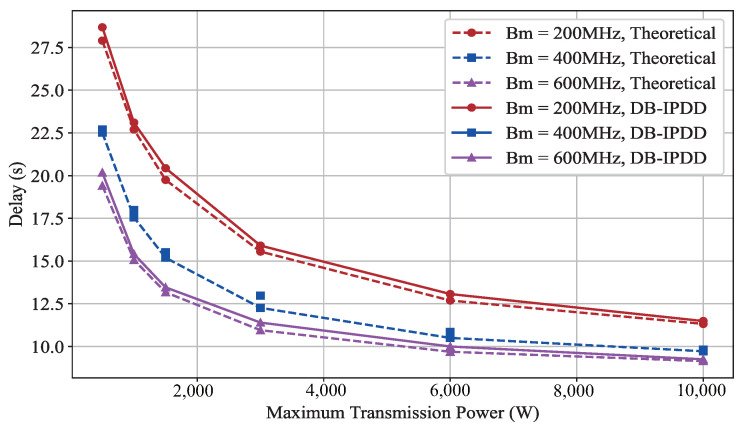
The delay performance with different transmit power: simulation and ideal delay curves under different bandwidth.

We assume that there are four SSs, each positioned at a distance of $5\times 10^{5}$ m from the MS. We alter the maximum transmit power of the MS by 500 W, 1000 W, 1500 W, 3000 W, 6000 W, 10,000 W] and the bandwidth by [200 MHz, 400 MHz, 600 MHz], observing their impact on the maximum time delay, as shown in Figure 5. The horizontal axis represents the number of iterations, and the vertical axis represents the delay in seconds.

As can be observed from Figure 5, it is easy to see that the curves show a downward trend with increasing power. However, with the increase in power, the decrease in the delay value is not obvious. This is because the relationship between power and delay is not linear but follows a diminishing reward. This conclusion is important from the engineering perspective because we need a moderate power setting that is cost-effective to achieve the trade-off between power and delay.

### 5.3. Delay Performance with Different SSs and Transmit Power

**Figure 6:**
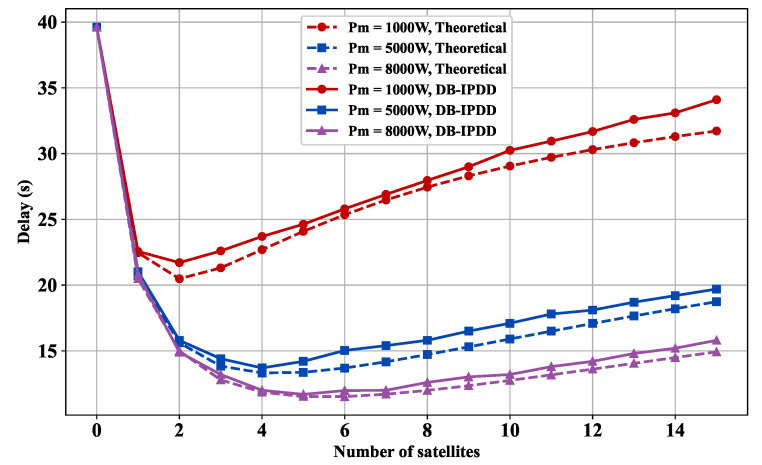
Delay versus satellite number: simulation and ideal delay curves under different transmission powers.

Then, we vary the number of SSs and keep the other variables constant. As shown in Figure 6, the delay performance is shown with different numbers of SSs and transmit power. It can be observed that the curve initially descends and then ascends after temporarily stabilizing as the number of satellites increases, which means that more SSs actually constrain the delay performance. This is because the available bandwidth is shared among all the SSs, and the bandwidth assigned to each SS is reduced as the number of SSs increases, resulting in a growing competition for resources. Therefore, it is important to carefully manage the number of SSs in order to maintain optimal system performance.

In Figure 6, we also choose different transmission powers [1000 W, 5000 W, 8000 W] and compare them with the relationship curve between the calculated time delay and the number of SSs derived from theoretical analysis. The delay decreases as the transmission power increases, given the same number of SSs.

### 5.4. Delay Performance with Different SSs and Bandwidths

**Figure 7:**
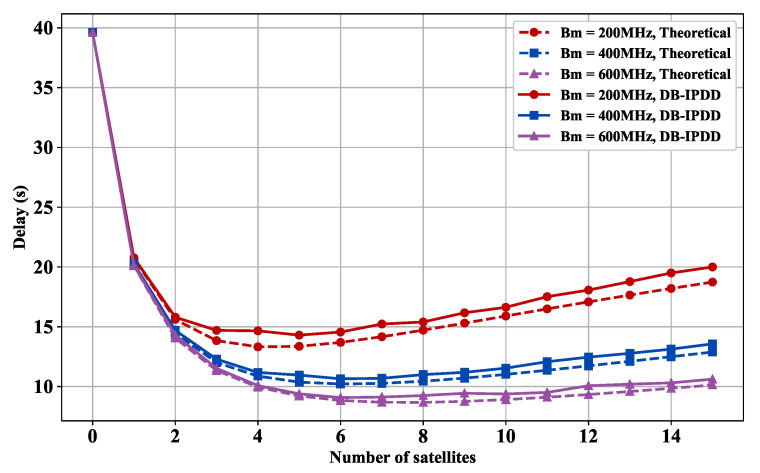
Delay versus satellite number: simulation and ideal delay curves under different bandwidths.

As shown in Figure 7, we choose different bandwidths from [200 MHz, 400 MHz, 600 MHz] and observe how the delay varies with the number of satellites in different bandwidths. Figure 7 shows that each 300 MHz bandwidth increase reduces data transmission delay by 22.5%.

### 5.5. Delay Performance with Different SSs and Computation Resources

We choose different main frequencies from [300 MHz, 400 MHz, 500 MHz] and compute the delay with the different number of SSs.

**Figure 8:**
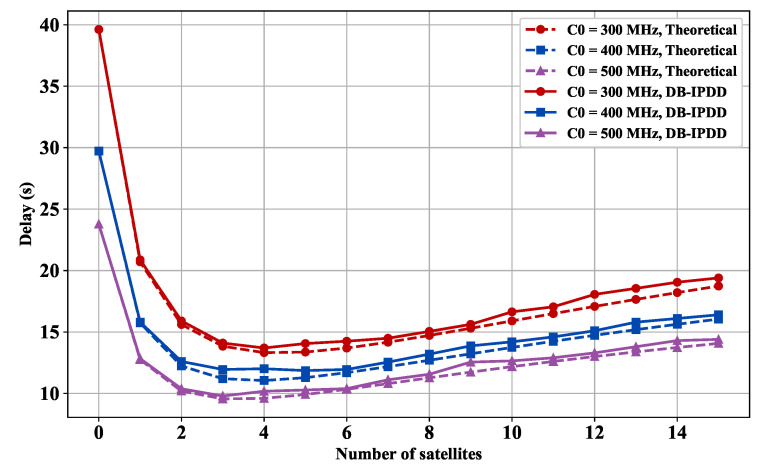
Delay versus satellite number: simulation and ideal delay curves under different main frequency.

Figure 8 demonstrates that each 100 MHz computing resource boost decreases processing delay by 16.62%. However, after a certain point, a further increase in bandwidth or satellite number does not significantly reduce the delay.

Figure 7 and Figure 8 reveal a non-monotonic relationship between the onboard computing node scale and the delay under dual constraints of total system power and bandwidth. As edge computing satellites increase from initial numbers, system delay first decreases then increases.

With fewer than four satellites, distributed parallel processing reduces delay. Beyond this threshold, constrained bandwidth linearly reduces per-satellite channel capacity, increasing transmission delay. Concurrently, total power constraints force reduced computing power per node, elevating processing delay. Numerical simulations show that exceeding four nodes creates a coupling effect between transmission and processing delays, causing inflection point and diminishing marginal returns. The optimal configuration is four satellites.

### 5.6. Delay Performance with Different Computation Resources and Different Bandwidths

**Figure 9:**
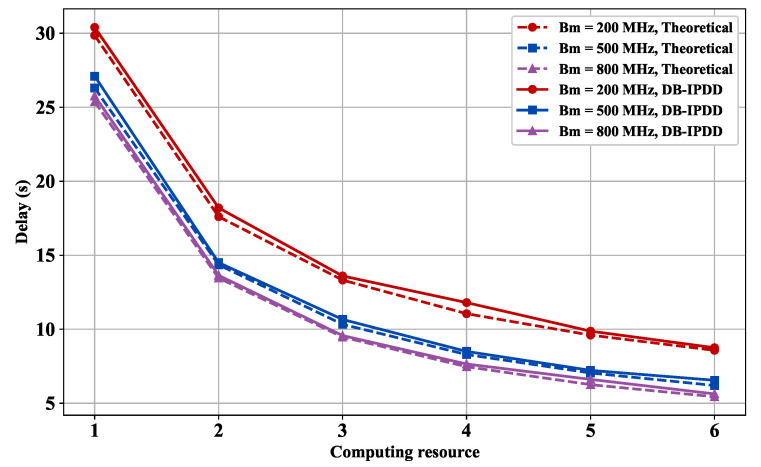
Delay versus main frequency: simulation and ideal delay curves under different bandwidths.

We choose different bandwidths from [200 MHz, 500 MHz, 800 MHz] and compute the delay under the condition of different main frequencies. The result is shown in Figure 9, which indicates that a 4 kW elevation in transmission power threshold achieves 24.89% total delay reduction, confirming the critical relationship between power configuration and latency performance.

### 5.7. Delay Performance with Different Main Frequencies and Transmission Powers

**Figure 10:**
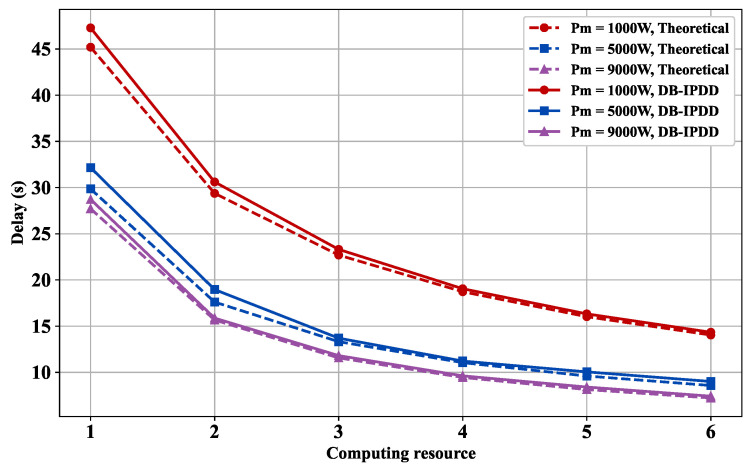
Delay versus main frequency: simulation and ideal delay curves under different transmission powers.

We choose different transmission powers from [1000 W, 5000 W, 9000 W] and observe the delay with different main frequencies. Figure 10 reveals that computation resources expansion effectively alleviates inter-satellite communication bottlenecks, showing a 13.2% data transmission latency reduction per 300 MHz main frequency increase.

Figure 9 and Figure 10 demonstrate an inverse correlation between system latency and computing resource allocation under dual constraints of total power and bandwidth. Enhanced computing capability reduces single-node processing latency while intelligent task allocation optimizes workload distribution, collectively lowering system latency when bandwidth remains constant.

### 5.8. Delay Performance in General Conditions

**Figure 11:**
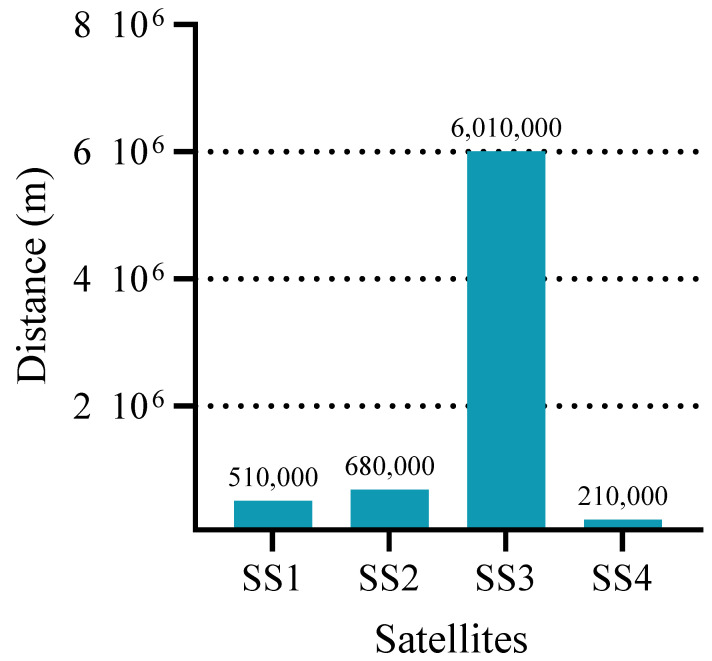
The distances of each SS.

**Figure 12:**
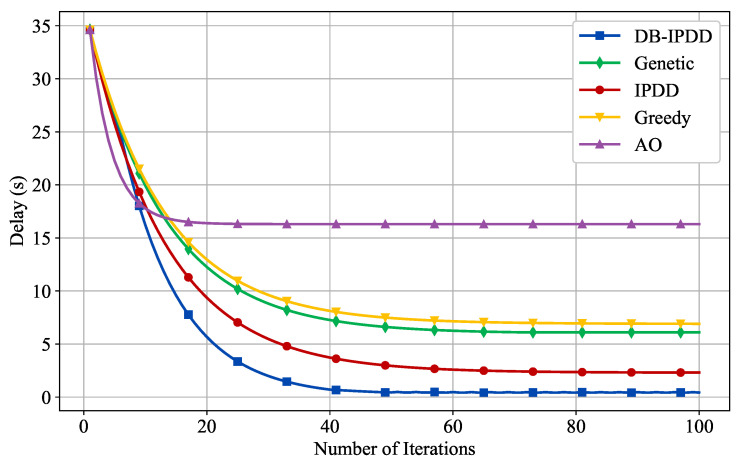
The delay of different schemes with varying number of iterations in a general scenario.

Indeed, the distances between the SSs and the MS are not necessarily similar and can vary significantly. To further validate the performance of the proposed DB-IPDD algorithm in a scenario closer to real-world conditions, we assume the distances are as follows: [5.1,6.8,60.1,2.1] × 10^5^ m, in which a significantly large value exists, which is shown in Figure 11. The convergence curve is shown in Figure 12, and we can see that the proposed algorithm is able to converge after 30 iterations in a relatively extreme scenario and the DB-IPDD method obtains a better performance than the IPDD method.

**Figure 13:**
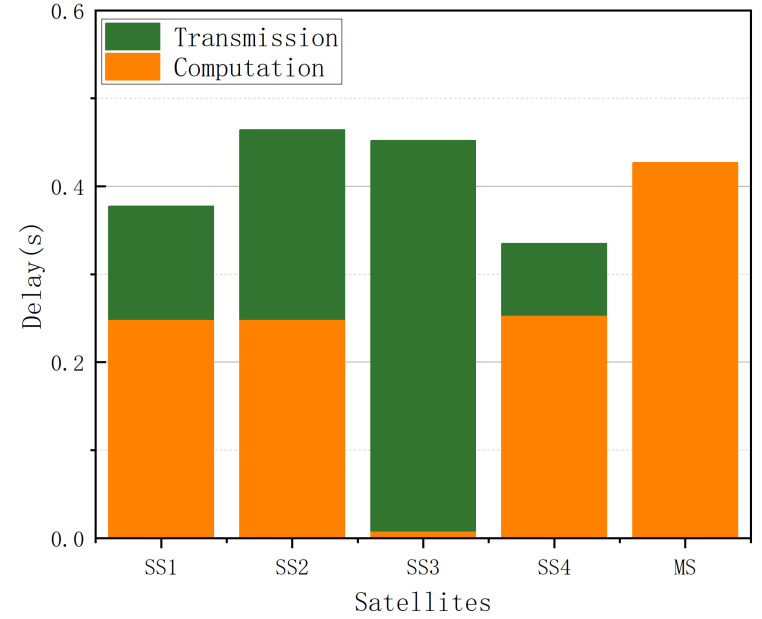
The delay in each SS.

The delay performance of each SS and the MS is plotted in the bar chart in Figure 13. We can conclude that the proposed algorithm is able to find the optimal solution, even in a general relatively extreme scenario, because the approximate delay value among all the satellites meets the conclusion of Theorem 1.

**Figure 14:**
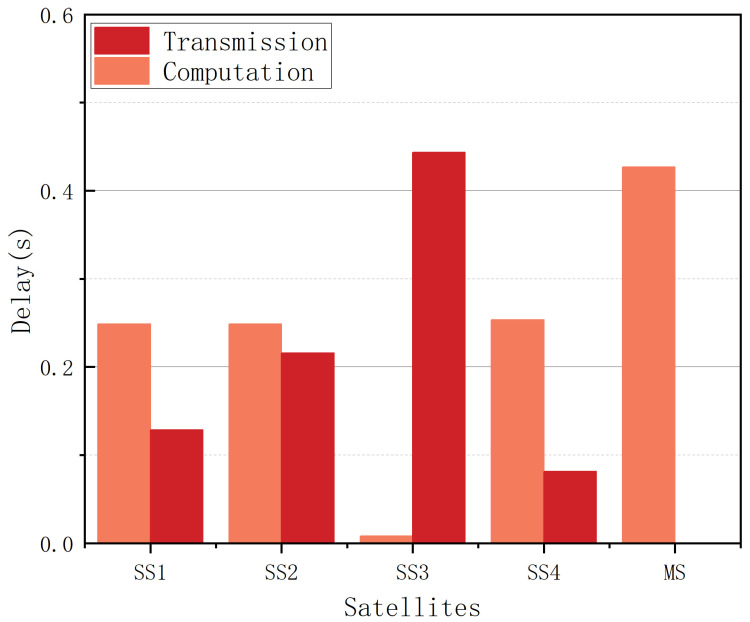
The computation delay and transmission delay in each SS.

Based on our analysis, the procedure can be divided into two parts, i.e., transmission and computation. The comparison in each satellite is shown in Figure 14. Generally speaking, as the distance increases, the computation delay tends to decrease while the transmission delay increases, and the reverse holds when the distance decreases.

## 6. Conclusions

In this paper, we consider the scenario of a LEO satellite edge computing system with time-sensitive tasks. We formulate an optimization problem to minimize the total computing and transmitting delay of the system, present Theorem 1 theoretically, and propose a joint allocation strategy of transmission power and computing resources to handle the challenging nonconvex problem. The convergence performance is validated in the simulation, and the delay performance is demonstrated under various conditions, both of which demonstrate the significant advantages of the proposed DB-IPDD algorithm by leveraging the feature of distributed balance. In future work, we plan to investigate intelligent computation offloading strategies in integrated space-terrestrial networks.

## Figures and Tables

**Table 1:** Abbreviations.

Abbreviations	Description
N	Number of slots
M	Number of slave satellites
$E_{1}$	Energy upper limit to execute the whole task
$E_{2}$	Energy upper limit of a satellite
D	Data needs to be processed
$C_{0}$	The main frequency of the CPU
$R_{k,i}$	The normalized transmission data rate
$\sigma^{2}$	The receiver noise power
B	Communication bandwidth
**Notation**	**Description**
c	The light speed
$r_{0}$	The Chip’s coefficient
*s*	Cycles needed to process 1 bit of data
η	The upper limit of the violation function
$d_{k,i}$	The distance between the *k*-th slave satellite
	and the master satellite
$\alpha_{k,i}$	The ratios of the offloading data to slave satellite
	*k* at slot *i*
$t_{k,i}$	The transmission delay to slave satellite
	*k* at slot *i*
$t_{0,i}$	The local computing time
$\mu_{0}$	The channel power gain at d = 1 m
λ,ρ	The Lagrange multiplier
$P_{k,i}$	The transmission power to slave satellite
	*k* at slot *i*

**Table 2:** Simulation parameters.

Parameter	Default Value
B	200 MHz
M	4
$C_{0}$	$3\;\times \;10^{8}$ cycles/s
$P_{m}$	5000 W
$E_{1}$	3,000,000 J
$\sigma^{2}$	1 $\times \;10^{-8}$ W
s	0.1
D	1 GB
γ	0.01
IterationNumber	200
$\mu_{0}$	1
λ,ρ	1

## Data Availability

No new data were created or analyzed in this study. Data sharing is not applicable to this article.

## Appendix A

### Appendix A.1. Proof of Theorem 1

**Lemma** **A1.** 
*If there is a disparity in delay between two satellites, we can reduce the large delay and increase the small delay by reallocating resources, which ultimately leads to the same delay for both satellites.*

**Proof.** According to the expression of delay (9), (10), it can be observed that delay increases monotonically with the allocation ratio of satellites and decreases monotonically with the transmission power. Assuming there exist two satellites named Satellite 1 and Satellite 2, and the delay of each is named as $t_{1}$ and $t_{2}$, where $t_{1}\geq t_{2}$, we can achieve $t_{1}^{*}=t_{2}^{*}$ by implementing one or both of the following actions:

1. Transfer a number of data from **Satellite 1** to **Satellite 2**: by shifting some data processing tasks from **Satellite 1** to **Satellite 2**, we can reduce the load and processing time on **Satellite 1**, potentially decreasing $t_{1}$ and increasing $t_{2}$.
2. Transfer a portion of power from **Satellite 2** to **Satellite 1**: by reallocating power resources from **Satellite 2** to **Satellite 1**, we can enhance the performance and transmission capability of **Satellite 1**, potentially reducing $t_{1}$ and multiplying $t_{2}$.

□

Suppose that when the system reaches minimum delay in a fixed time slot, the delays of individual satellites are not identical. There exists a set A satisfying

$$t_{j}=S=\max\left\{t_{0,i},t_{1,i},\ldots ,t_{M,i}\right\}\;\forall j\in A$$

Correspondingly, there exists a set B where

$$t_{k}<S\;\forall k\in B$$

According to Lemma A1, we can allocate resources by pairing any $t_{j,i}$ from set A with any $t_{k,i}$ from set B. This yields the reallocated relationship:

$$t_{j,i}\geq t_{j,i}^{*}=t_{k,i}^{*}\geq t_{k,i}$$

where $t_{j,i}^{*}$ and $t_{k,i}^{*}$ denote the updated delays after resource reallocation. Extending this process to all satellites in A, we obtain:

$$t_{k}\geq t_{k}^{*}\;\forall k\in A$$

Define the new maximum delay:

$$S^{*}=\max\left\{t_{0,i}^{*},t_{1,i}^{*},\ldots ,t_{M,i}^{*}\right\}$$

It necessarily follows that:

$$S^{*}\leq S$$

This contradiction implies *S* cannot be the minimum delay. Therefore, when the system achieves minimum delay in a fixed slot, all satellite delays must satisfy:

$$t_{0,i}=t_{1,i}=\cdots =t_{M,i}$$

## References

[1] Xia Q., Wang G., Xu Z., Liang W., Xu Z. (2024). Efficient Algorithms for Service Chaining in NFV-Enabled Satellite Edge Networks. IEEE Trans. Mob. Comput..

[2] Zhang H., Liu R., Kaushik A., Gao X. (2023). Satellite Edge Computing with Collaborative Computation Offloading: An Intelligent Deep Deterministic Policy Gradient Approach. IEEE Internet Things J..

[3] Ji Z., Wu S., Jiang C. (2023). Cooperative Multi-Agent Deep Reinforcement Learning for Computation Offloading in Digital Twin Satellite Edge Networks. IEEE J. Sel. Areas Commun..

[4] Wang Y., Yang J., Guo X., Qu Z. (2020). A game-theoretic approach to computation offloading in satellite edge computing. IEEE Access.

[5] Yousefpour A., Fung C., Nguyen T., Kadiyala K., Jalali F., Niakanlahiji A., Kong J., Jue J.P. (2019). All one needs to know about fog computing and related edge computing paradigms: A complete survey. J. Syst. Archit..

[6] Zhang H., Zhao H., Liu R., Gao X., Xu S. (2024). Leader Federated Learning Optimization Using Deep Reinforcement Learning for Distributed Satellite Edge Intelligence. IEEE Trans. Serv. Comput..

[7] Zhang Y., Zhang H., Sun K., Huo J., Wang N., Leung V.C.M. (2024). Partial Computation Offloading in Satellite-Based Three-Tier Cloud-Edge Integration Networks. IEEE Trans. Wirel. Commun..

[8] Zhou J., Zhao Y., Zhao L., Cai H., Xiao F. (2024). Adaptive Task Offloading with Spatiotemporal Load Awareness in Satellite Edge Computing. IEEE Trans. Netw. Sci. Eng..

[9] Chen D., Liu Y.C., Kim B.G., Xie J., Hong C.S., Han Z. (2020). Edge computing resources reservation in vehicular networks: A meta-learning approach. IEEE Trans. Veh. Technol..

[10] Pham Q.V., Ruby R., Fang F., Nguyen D.C., Yang Z., Le M., Ding Z., Hwang W.J. (2022). Aerial Computing: A New Computing Paradigm, Applications, and Challenges. IEEE Internet Things J..

[11] Li Y., Gao X., Shi M., Kang J., Niyato D., Yang K. (2025). Dynamic Weighted Energy Minimization for Aerial Edge Computing Networks. IEEE Internet Things J..

[12] Letaief K.B., Shi Y., Lu J., Lu J. (2022). Edge Artificial Intelligence for 6G: Vision, Enabling Technologies, and Applications. IEEE J. Sel. Areas Commun..

[13] Fang X., Feng W., Wei T., Chen Y., Ge N., Wang C.-X. (2021). 5G Embraces Satellites for 6G Ubiquitous IoT: Basic Models for Integrated Satellite Terrestrial Networks. IEEE Internet Things J..

[14] Hassan S.S., Park Y.M., Tun Y.K., Saad W., Han Z., Hong C.S. (2024). Satellite-Based ITS Data Offloading & Computation in 6G Networks: A Cooperative Multi-Agent Proximal Policy Optimization DRL with Attention Approach. IEEE Trans. Mob. Comput..

[15] Xie R., Tang Q., Wang Q., Liu X., Yu F.R., Huang T. (2020). Satellite-Terrestrial Integrated Edge Computing Networks: Architecture, Challenges, and Open Issues. IEEE Netw..

[16] Shi Q., Hong M. (2020). Penalty Dual Decomposition Method for Nonsmooth Nonconvex Optimization—Part I: Algorithms and Convergence Analysis. IEEE Trans. Signal Process..

[17] Shi Q., Hong M., Fu X., Chang T.-H. (2020). Penalty Dual Decomposition Method for Nonsmooth Nonconvex Optimization—Part II: Applications. IEEE Trans. Signal Process..

[18] Li Q., Wang S., Ma X., Zhou A., Wang Y., Huang G., Liu X. (2024). Battery-Aware Energy Optimization for Satellite Edge Computing. IEEE Trans. Serv. Comput..

[19] Wu J., Jia M., Guo Q., Gu X. Joint Optimization Computation Offloading and Resource Allocation for LEO Satellite with Edge Computing. Proceedings of the 2023 IEEE International Symposium on Broadband Multimedia Systems and Broadcasting (BMSB).

[20] Song Z., Hao Y., Liu Y., Sun X. (2021). Energy-efficient multiaccess edge computing for terrestrial-satellite internet of things. IEEE Internet Things J..

[21] Zhang Y., Chen C., Liu L., Lan D., Jiang H., Wan S. (2022). Aerial edge computing on orbit: A task offloading and allocation scheme. IEEE Trans. Netw. Sci. Eng..

[22] Yi C., Cai J., Su Z. (2020). A multi-user mobile computation offloading and transmission scheduling mechanism for delay-sensitive applications. IEEE Trans. Mob. Comput..

[23] Chen X., Jiao L., Li W., Fu X. (2016). Efficient multi-user computation offloading for mobile-edge cloud computing. IEEE Trans. Netw..

[24] Zhang X., Liu J., Zhang R., Huang Y., Tong J., Xin N., Liu L., Xiong Z. (2024). Energy-Efficient Computation Peer Offloading in Satellite Edge Computing Networks. IEEE Trans. Mob. Comput..

[25] Zhou J., Yang Q., Zhao L., Dai H., Xiao F. (2024). Mobility-Aware Computation Offloading in Satellite Edge Computing Networks. IEEE Trans. Mob. Comput..

[26] Kim T., Kwak J., Choi J.P. (2022). Satellite Edge Computing Architecture and Network Slice Scheduling for IoT Support. IEEE Internet Things J..

[27] Qiu C., Yao H., Yu F.R., Xu F., Zhao C. (2019). Deep Q-learning aided networking, caching, and computing resources allocation in softwaredefined satellite-terrestrial networks. IEEE Trans. Veh. Technol..

[28] Hu Q., Cai Y., Yu G., Qin Z., Zhao M., Li G.Y. (2019). Joint Offloading and Trajectory Design for UAV-Enabled Mobile Edge Computing Systems. IEEE Internet Things J..

